# An Unusual Vascular Catastrophe: Atheromatous Aortic Dissection With Renal Artery Thrombosis and Cortical Laminar Necrosis Post Cerebral Ischemia

**DOI:** 10.7759/cureus.94891

**Published:** 2025-10-18

**Authors:** Sona Murugan, Praveenraja Shanmugam, Khin Nyo, Naing Htoon

**Affiliations:** 1 General Internal Medicine, Doncaster and Bassetlaw NHS Foundation Trust, Doncaster, GBR; 2 General Medicine, North West Anglia NHS Foundation Trust, Peterborough, GBR; 3 Internal Medicine/Stroke, Doncaster and Bassetlaw NHS Foundation Trust, Doncaster, GBR; 4 Internal Medicine/Stroke, Sheffield Teaching Hospitals NHS Foundation Trust, Sheffield, GBR

**Keywords:** abdominal aortic dissection, acute kidney injury, cortical laminar necrosis, ischemic kidney injury, renal artery thrombosis

## Abstract

Spontaneous atheromatous aortic dissection is a rare but serious vascular complication caused by the rupture of an atherosclerotic plaque, leading to distal vessel occlusion and ischemic injury such as acute renal infarction. Cortical laminar necrosis (CLN) represents a permanent form of cerebral cortical damage following severe ischemia, characterized by selective neuronal necrosis and distinct radiological features, and is associated with poor neurological prognosis.

We report the case of a 67-year-old woman with atrial fibrillation, heart failure, peripheral vascular disease, and a heavy smoking history of 40 pack-years, who presented with sudden expressive dysphasia and right-sided weakness. Initial brain computed tomography (CT) demonstrated a subacute left frontal infarction, and follow-up imaging confirmed cortical laminar necrosis.

During admission, she developed abrupt anuria and stage 3 acute kidney injury without abdominal pain. CT angiography revealed the spontaneous atheromatous dissection of the abdominal aorta below the superior mesenteric artery, with the complete thrombosis of the right renal artery causing acute infarction, and a chronically atrophic left kidney from long-standing ischemia. In view of multiorgan involvement and poor prognosis, a multidisciplinary decision was made for palliative care.

This case illustrates the occurrence of two uncommon but severe vascular complications in a patient with significant cardiovascular risk factors: spontaneous atheromatous dissection leading to renal artery thrombosis (RAT) and cortical laminar necrosis following ischemic stroke. Both conditions reflect the systemic burden of advanced atherosclerosis and underscore the importance of early recognition, prompt imaging, and a multidisciplinary approach to guide management in high-risk patients.

## Introduction

The spontaneous atheromatous dissection of the abdominal aorta is an uncommon but clinically significant vascular event. Unlike classical aortic dissection, which typically arises from an intimal tear, atheromatous dissection results from the rupture of a severely diseased aortic plaque. This can lead to a penetrating atherosclerotic ulcer that extends through the elastic lamina, with hematoma formation in the aortic media, potentially causing catastrophic ischemic complications such as acute renal infarction [1].

Cortical laminar necrosis (CLN) represents another severe manifestation of vascular and metabolic compromise in the brain. It is characterized by the delayed, selective necrosis of the cerebral cortex, predominantly the third cortical layer, with greater involvement of sulcal depths compared to gyral crests [2,3]. CLN has been described in association with hypoxic-ischemic encephalopathy, hypoglycemia, prolonged seizures, watershed infarctions, metabolic and autoimmune disorders, and after immunosuppressive or antineoplastic therapy. Although uncommon, CLN is generally considered a marker of poor neurological outcome, with functional recovery influenced by cerebral metabolism, perfusion, and inflammatory responses [4]. In our patient, follow-up computed tomography (CT) imaging nine days after stroke confirmed CLN, which correlated with persistent expressive dysphasia and right-sided weakness, highlighting the prognostic significance of this lesion.

We present the case of a 67-year-old woman with advanced systemic atherosclerosis who developed cortical laminar necrosis after ischemic stroke, along with catastrophic renal infarction secondary to spontaneous atheromatous aortic dissection. This combination of severe cerebral and renal vascular complications in a single patient is exceptionally rare and highlights the systemic burden of advanced atherosclerosis. By documenting this case, we aim to raise awareness of these uncommon but potentially devastating complications and emphasize the importance of early recognition, prompt imaging, and a multidisciplinary approach in high-risk patients.

## Case presentation

**Figure 1:**
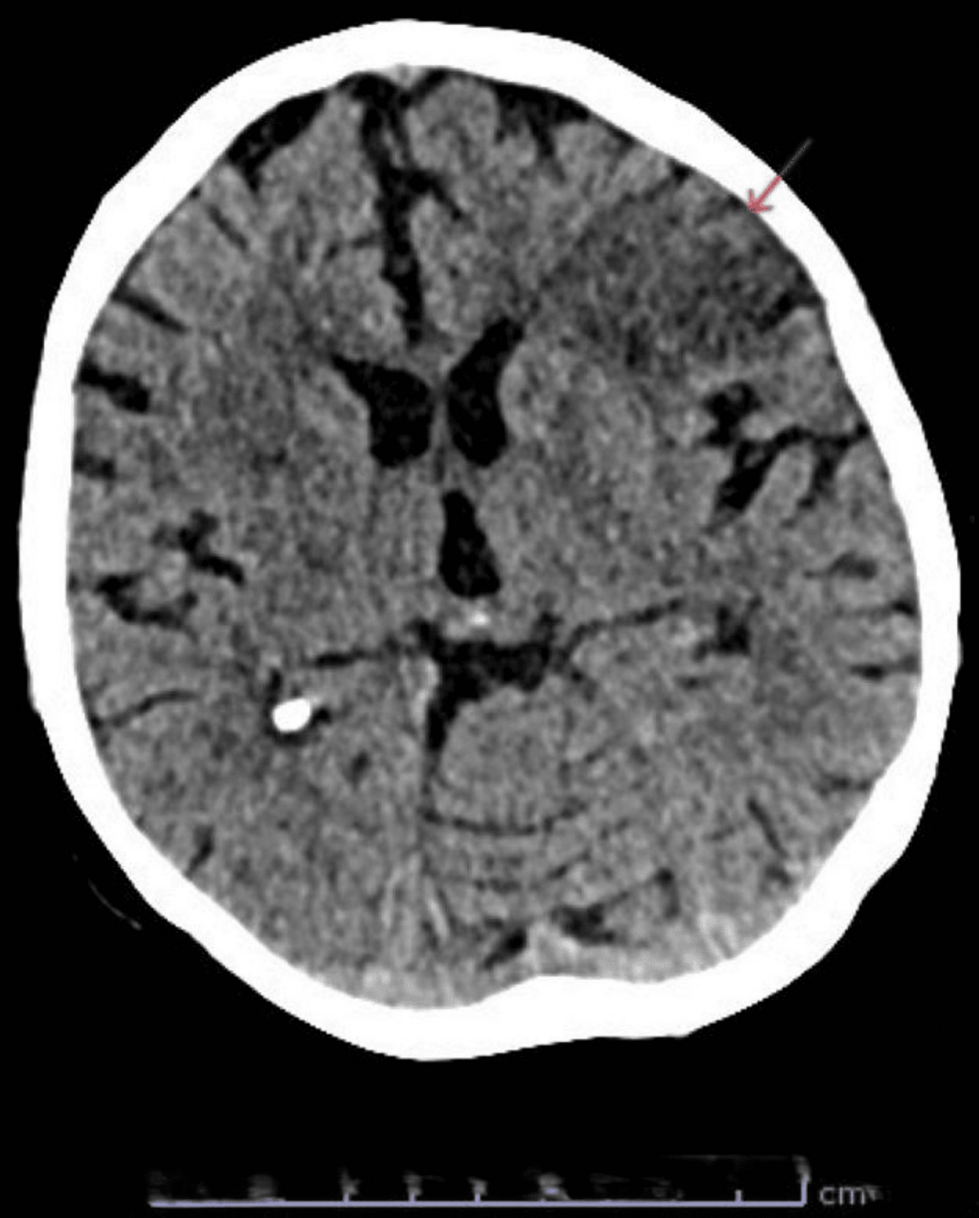
Initial non-contrast computed tomography of the head demonstrating a subacute infarct in the left frontal lobe, seen as hypodensity with a mild loss of gray-white matter differentiation (area of interest marked by arrow), corresponding to the patient’s presenting expressive dysphasia and right-sided weakness.

**Figure 2:**
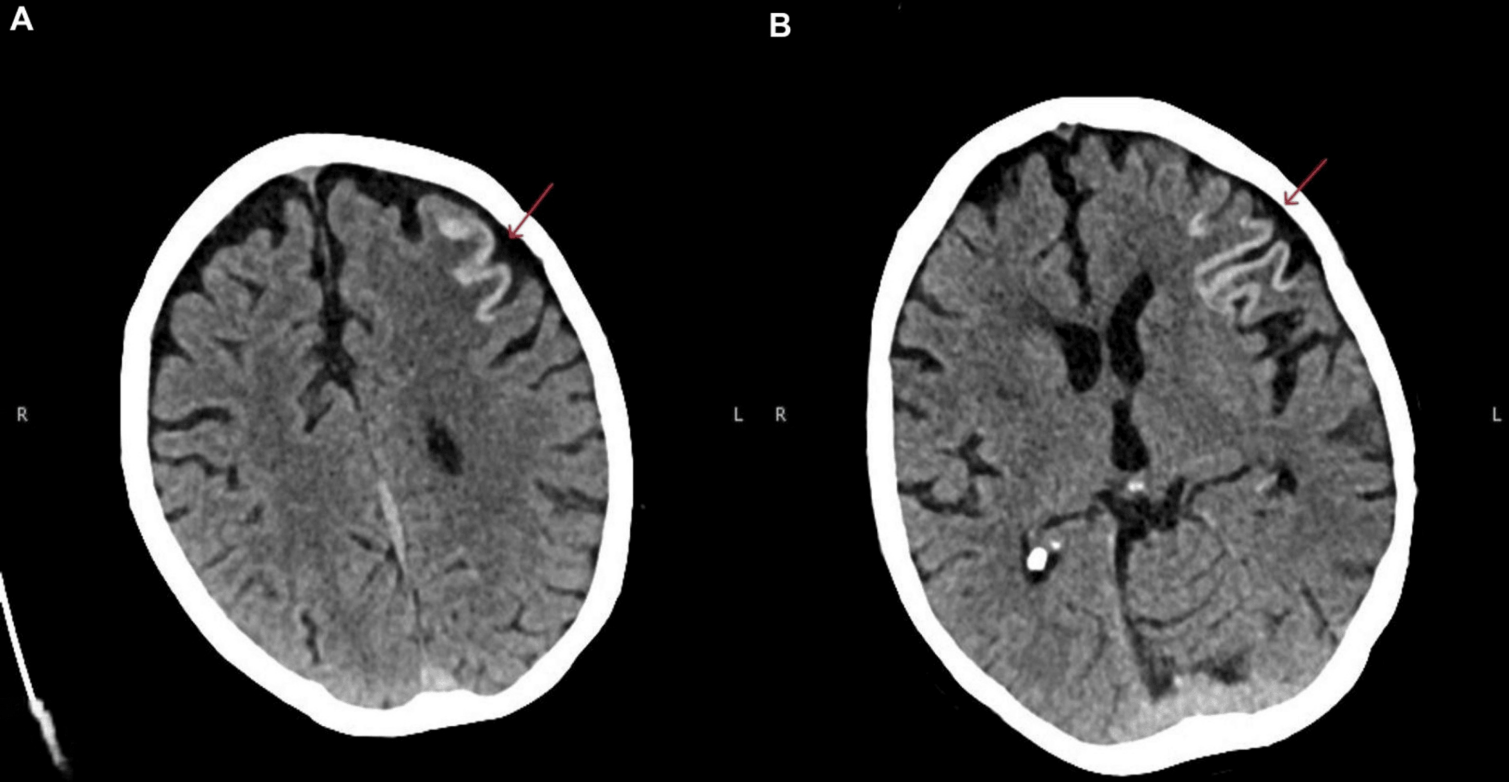
Axial non-contrast computed tomography (CT) of the head showing cortical laminar necrosis in the left frontal lobe, with hyperattenuation along the cortical ribbon consistent with irreversible ischemic neuronal injury.

A 67-year-old woman with a history of atrial fibrillation on apixaban, heart failure, peripheral vascular disease with bilateral iliac artery stenosis, and chronic heavy smoking (>40 pack-years) presented with a three-day history of expressive dysphasia and right-sided weakness. On admission, her blood pressure was 160/90 mmHg. Baseline electrocardiogram (ECG) showed atrial fibrillation with a controlled ventricular rate and no new ischemic changes, consistent with her known cardiac history. Initial non-contrast CT of the head demonstrated a subacute left frontal infarct (Figure 1), and carotid Doppler revealed bilateral internal carotid artery stenosis of approximately 50%. She was started on aspirin 300 mg and admitted to the hyperacute stroke unit. A follow-up CT of the head was performed around nine days after initial stroke presentation, to guide the optimization of anticoagulation treatment, which revealed cortical laminar necrosis within the infarcted region (Figure 2A, 2B), consistent with severe ischemic injury.

**Figure 3:**
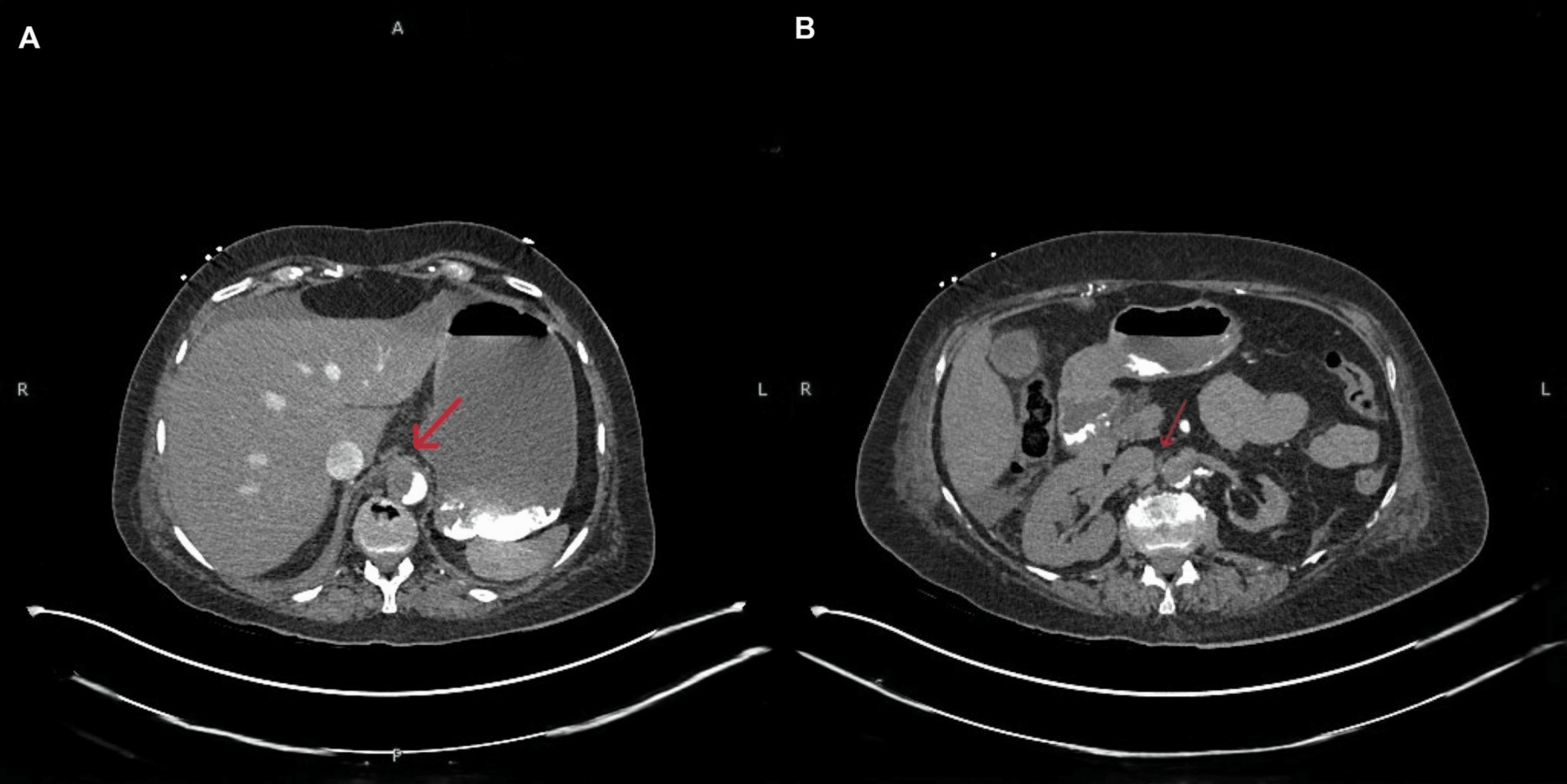
Computed tomography (CT) of the abdomen showing an atheromatous dissection and occlusion of the abdominal aorta below the level of the superior mesenteric artery (SMA), with the complete occlusion of the right renal artery due to the acute thrombosis and near-total infarction of the right renal parenchyma. The left kidney appears markedly atrophic with chronic ischemic changes, attributable to long-standing proximal left renal artery occlusion.

During admission, the patient developed abrupt anuria and stage 3 acute kidney injury (Table 1) initially without abdominal pain or other visceral symptoms. Persistent elevated lactate despite the correction of pre-renal causes prompted further imaging. CT of the abdomen demonstrated an atheromatous dissection and occlusion of the abdominal aorta below the superior mesenteric artery (Figure 3A), the complete thrombosis of the right renal artery with near-total right renal infarction, and a markedly atrophic left kidney with chronic ischemic changes (Figure 3B).

**Table 1:** Blood Investigations.

Parameters	Day 1	Day 3	Day 4	Reference range
Urea	4.1	13.7	17.8	2.5-7.8 mmol/L
Creatinine	93	407	503	64-104 µmol/L
Lactate	2.1	5.7	7.9	0.50-2.20 mmol/L

A multidisciplinary team (MDT) approach involving vascular and renal teams was undertaken. In view of multiorgan involvement, the treatment limitation of reversible conditions, and poor prognosis, it was decided not to pursue further intervention, and a best interest decision was made for palliation. Afterward, patient care was focused on comfort measures and supportive management.

## Discussion

Spontaneous atheromatous dissection of the aorta with renal artery thrombosis (RAT)

This case highlights the spontaneous atheromatous dissection of the abdominal aorta, which is an uncommon but clinically significant vascular event. Unlike classical aortic dissection, which typically arises from an intimal tear, atheromatous dissection results from the rupture of a severely diseased aortic plaque, leading to a penetrating atherosclerotic ulcer that extends through the elastic lamina with hematoma formation in the aortic media [1]. This process causes dissection and is pathologically distinct from both classical dissection and frank aortic rupture.

Aortic dissection can also involve branch vessels, further complicating clinical presentation. Dissections are classified as Stanford Type A (involving the ascending aorta) or Type B (distal to the left subclavian artery), with Type B dissections frequently managed medically through blood pressure control [5]. Clinical manifestations vary widely and may include limb ischemia, syncope, stroke, acute renal failure, mesenteric ischemia, myocardial infarction, or back and abdominal pain. While pain is commonly reported, clinicians should consider aortic dissection even in its absence, as demonstrated in our patient.

These lesions can result in distal thromboembolic events, including acute renal artery thrombosis (RAT) as observed in our case. RAT is often under-recognized because its symptoms are nonspecific and can mimic conditions such as renal colic or other abdominal pathologies [6]. Atheromatous plaques contain cholesterol crystals and inflammatory material. Plaque rupture can release embolic fragments that occlude downstream vessels, causing organ ischemia. Cholesterol crystals can be detected in blood using polarized light microscopy, reflecting ongoing plaque instability. The literature suggests that up to 5% of aortic plaques may result in clinically detectable thromboembolic events [7].

Renal artery thrombosis is a rare cause of acute kidney injury. Postmortem studies suggest that it occurs in up to 1.4% of the general population, though less than 1% are symptomatic. Among patients presenting to the emergency department, the incidence of renal infarction is estimated to be <0.007%, with bilateral involvement in approximately 16% of cases. The renal artery is the least common site for thrombosis (<2%), compared with peripheral arteries (61%), mesenteric arteries (29%), pelvic arteries (9%), and the aorta (7%). Most patients with acute kidney injury have pre-renal or intrinsic causes, with renal artery thrombosis accounting for only ~1% of cases [8].

Our patient developed abrupt anuria and stage 3 acute kidney injury without abdominal pain, highlighting an unusual presentation in high-risk patients. Acute renal artery occlusion (RAO) remains diagnostically challenging, with potential clinical features including flank pain, hematuria, uncontrolled hypertension, fever, or acute renal failure [9]. Prompt imaging, particularly CT angiography, is crucial for diagnosis and management planning.

Cortical laminar necrosis after ischemic stroke

Cortical laminar necrosis (CLN) is a distinct pattern of permanent cortical injury characterized by the delayed, selective necrosis of the cerebral cortex, most prominently affecting the third cortical layer. The injury is usually greater in the sulcal depths than the gyral crests and involves neurons, glial cells, and blood vessels, producing a band-like pattern of pan-necrosis visible in late subacute or chronic stages of energy failure [2,3]. CLN has been reported in association with hypoxic-ischemic encephalopathy, watershed infarctions, hypoglycemia, status epilepticus, metabolic disorders, autoimmune conditions, and after immunosuppressive or antineoplastic therapy [4]. CLN is usually regarded as a marker of poor neurological outcome; functional recovery is not uniform and is influenced by multiple factors, including cerebral metabolism, immune and inflammatory responses, and perfusion status.

The pathophysiology relates to the critically prolonged impairment of cerebral metabolism and energy supply, with the higher metabolic demand of gray matter rendering it particularly vulnerable. Imaging findings are characteristic: hyperdense gyriform lesions on CT and cortical T1 hyperintensity on magnetic resonance imaging (MRI), which reflect protein denaturation and lipid-laden macrophage accumulation rather than hemorrhage or calcification [2,10]. These T1 hyperintensities typically emerge 3-5 days after infarction, peak at around one month, and gradually fade but may persist for over a year [10]. In our patient with significant cardiovascular risk factors, CLN was identified on follow-up CT nine days after stroke, performed to guide anticoagulation, confirming the severity of ischemic cortical injury.

## Conclusions

This case highlights the rare coexistence of renal artery thrombosis due to spontaneous atheromatous aortic dissection and cortical laminar necrosis following ischemic stroke in a patient with extensive cardiovascular comorbidities. Renal artery thrombosis, although uncommon, should be considered a differential diagnosis in acute kidney injury, particularly when conventional pre-renal or intrinsic causes are insufficient to explain the clinical course. Cortical laminar necrosis, meanwhile, serves as an imaging biomarker of severe ischemic cortical injury and portends an unfavorable neurological prognosis.

The timely recognition of both conditions requires a high index of suspicion and reliance on advanced imaging modalities. Given the complexity and multiorgan involvement, optimal management depends on early diagnosis; the integration of vascular, neurological, and renal expertise; and individualized treatment strategies.
